# Evaluation of Hydroxyapatite–β-Tricalcium Phosphate Collagen Composites for Socket Preservation in a Canine Model

**DOI:** 10.3390/jfb16080286

**Published:** 2025-08-03

**Authors:** Dong Woo Kim, Donghyun Lee, Jaeyoung Ryu, Min-Suk Kook, Hong-Ju Park, Seunggon Jung

**Affiliations:** Department of Oral and Maxillofacial Surgery, School of Dentistry, Dental Science Research Institute, Chonnam National University, 33, Yongbong-ro, Buk-gu, Gwangju 61186, Republic of Korea; omsdwk@gmail.com (D.W.K.); lyk0161@gmail.com (D.L.); ryu@jnu.ac.kr (J.R.); omskook@jnu.ac.kr (M.-S.K.); omspark@jnu.ac.kr (H.-J.P.)

**Keywords:** alveolar ridge preservation, beta-tricalcium phosphate, bone graft substitute, canine model, collagen composite, hydroxyapatite

## Abstract

This study aimed to compare the performance of three hydroxyapatite–β-tricalcium phosphate (HA–β-TCP) collagen composite grafts in a canine model for extraction socket preservation. Eight mongrel dogs underwent atraumatic bilateral mandibular premolar extraction, and sockets were randomly grafted with HBC28 (20% high-crystalline HA, 80% β-TCP bovine collagen), HBC37 (30% HA, 70% β-TCP, bovine collagen), or HPC64 (60% HA, 40% β-TCP, porcine collagen). Grafts differed in their HA–β-TCP ratio and collagen origin and content. Animals were sacrificed at 4 and 12 weeks, and the healing sites were evaluated using micro-computed tomography (micro-CT) and histological analysis. At 12 weeks, all groups showed good socket maintenance with comparable new bone formation. However, histological analysis revealed that HBC28 had significantly higher residual graft volume, while HPC64 demonstrated more extensive graft resorption. Histomorphometric analysis confirmed these findings, with statistically significant differences in residual graft area and bone volume fraction. No inflammatory response or adverse tissue reactions were observed in any group. These results suggest that all three HA–β-TCP collagen composites are biocompatible and suitable for socket preservation, with varying resorption kinetics influenced by graft composition. Selection of graft material may thus be guided by the desired rate of replacement by new bone.

## 1. Introduction

Adequate bone volume and quality are essential prerequisites for predictable success and long-term stability in dental implant procedures [1,2]. Clinical practice frequently encounters scenarios of insufficient alveolar bone volume and compromised bone quality due to trauma, periodontal disease, congenital anomalies, tumor resection, or prolonged edentulism [3,4]. These conditions significantly complicate implant treatment, increasing risks of inadequate implant stabilization and delayed osseointegration [5], and consequently prolonging the overall treatment duration. Bone grafting procedures have thus been developed and widely utilized to restore bone integrity, volume, and density [6]. Since a significant reduction in alveolar ridge volume often occurs following tooth extraction, socket preservation techniques have been introduced to prevent such resorption, and they are now widely employed in clinical practice. These techniques include using grafting materials such as autogenous bone, xenografts, allografts, and synthetic grafts such as hydroxyapatite (HA), β-tricalcium phosphate (β-TCP), and collagen composites.

Autologous bone has historically been considered the gold standard due to its osteogenic, osteoinductive, and osteoconductive properties [7,8]. However, despite its biological advantages, autologous bone is associated with several drawbacks, including limited availability, donor-site morbidity, prolonged surgery time, and increased postoperative discomfort [9,10,11]. These concerns have been consistently reported in both experimental and clinical studies and have led to an increasing shift toward synthetic and xenogeneic alternatives [12,13]. These alternative grafting materials must be capable of reliable bone regeneration without associated donor-site complications [14].

Synthetic and xenogeneic bone graft substitutes, notably β-TCP, have gained popularity due to their similarity to natural bone’s mineral composition, abundant availability, predictable degradation, and consistent biological performance [15,16]. These materials are frequently used in socket preservation procedures following tooth extraction, aiming to maintain alveolar bone volume and support successful subsequent implant placement.

However, β-TCP alone is highly resorbable and lacks the osteogenic potential of autografts, often requiring extended healing periods or additional biological enhancement [17]. In contrast, HA offers superior structural stability and slower resorption, but may persist longer in the defect site and delay complete remodeling. Consequently, composite materials have been developed to address the limitations of single-phase ceramics by integrating mineral scaffolds with collagen components—typically derived from bovine or porcine sources. While bovine-derived collagen is more commonly used, porcine collagen is also incorporated in some products. Although both types are clinically used, differences in their biological effects remain unclear, prompting the need for further investigation.

Composite graft materials integrating HA, β-TCP, and collagen have been developed to enhance biological performance [18,19]. Such combinations are believed to synergize the long-term stability of HA with the resorbability of β-TCP and the cellular compatibility of collagen, facilitating a balance between scaffold persistence and tissue integration [13,20]. Collagen facilitates osteoconduction and cellular proliferation and differentiation, improves osteoconductivity, and enhances biocompatibility [21,22,23]. Previous studies and reviews have reported improved bone regeneration outcomes using alloplastic graft materials, particularly those combining HA and β-TCP in varied ratios with collagen integration [24,25]. However, optimal ratios have yet to be clearly established. However, results remain inconsistent across studies, with some suggesting better outcomes at higher HA ratios [26], while others favor β-TCP-enriched scaffolds for faster remodeling [12]. Therefore, this study seeks to clarify how the relative proportions of HA, β-TCP, and collagen influence osteogenic performance in a controlled in vivo model.

This study evaluated three composite bone grafts: HBC28 (20% high-crystalline HA, 80% β-TCP, bovine collagen), HBC37 (30% HA, 70% β-TCP, bovine collagen), and HPC64 (60% HA, 40% β-TCP, porcine collagen). Unlike prior studies that focused on single material types or fixed HA/β-TCP ratios, this study compares clinically available composites with varying HA/β-TCP ratios and collagen sources under standardized extraction socket conditions in a canine model [12,26]. By analyzing torque measurements, implant stability, and histomorphometric outcomes, we aim to clarify how material composition affects bone regeneration dynamics. This study aims to guide graft selection for clinical applications and contribute new insights into the optimization of composite bone substitutes for socket preservation.

## 2. Materials and Methods

### 2.1. Animals

Eight healthy adult male mongrel dogs, averaging 15 kg in weight, were selected for this study. Animals were individually housed under standardized conditions. This study was approved by the Institutional Animal Care and Use Committee of Cronex (approval no. CRONEX-IACUC202301006) and conducted according to institutional ethical guidelines.

### 2.2. Experimental Design

**Figure 1:**
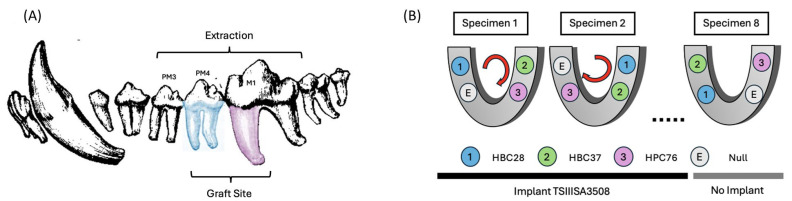
(**A**) Schematic illustration of the mandibular teeth involved in extraction and graft placement. The mandibular third premolar (PM3), fourth premolar (PM4), and the mesial root of the first molar (M1) were extracted to create standardized alveolar defects for bone grafting. (**B**) Rotational experimental design to minimize positional and anatomical variability. Each graft material (HBC28, HBC37, HPC64) and the empty control (E) were rotated systematically across different extraction sites within each animal to ensure even distribution and reduce potential anatomical bias.

A canine mandibular extraction socket model was established to simulate clinical conditions for socket preservation commonly encountered following tooth extraction in dental implant procedures. Bilateral mandibular third (PM3) and fourth (PM4) premolars, as well as the mesial roots of the mandibular first molars (M1), were extracted. These sites were chosen based on their anatomical consistency, accessibility, and frequent clinical involvement in implant planning. The assignment of graft materials to the sockets was systematically rotated across subjects and quadrants to minimize site-specific bias and achieve uniform distribution (Figure 1B).

### 2.3. Bone Substitutes

Three collagen-based composite bone graft materials with varying ratios of hydroxyapatite (HA) and β-tricalcium phosphate (β-TCP) were evaluated. Specifically, three commercially available products in Korea were selected based on their differing HA/β-TCP ratios: HBC28 (Q-Oss + Collagen; OSSTEM IMPLANT, Seoul, Republic of Korea), HBC37 (Osteon II Collagen; GENOSS, Suwon, Republic of Korea), and HPC64 (Osteon 3 Collagen; GENOSS, Suwon, Republic of Korea). The composition and morphological characteristics of each material are summarized in Table 1. All materials were clinically available in Korea at the time of the study.

Collagen was physically incorporated into the grafts as a surface coating or binder during the manufacturing process, enhancing scaffold cohesion and facilitating cellular attachment. According to the manufacturer, HBC28 was prepared by blending HA particles with a collagen fiber dispersion to achieve a final collagen content of approximately 10 wt%. In contrast, HBC37 and HPC64 contained approximately 4–6 wt% collagen, with bovine-derived type I collagen used in HBC37 and porcine-derived type I collagen in HPC64.

### 2.4. Surgical Procedures

All surgical interventions were performed under general anesthesia induced with Zoletil 50 (3–5 mg/kg) mixed with Xylazine-HCL (2.2 mg/kg). Anesthesia was maintained using 2–5% isoflurane with oxygen at 2 L/min. Local anesthesia with 2% lidocaine (5–10 mg) containing epinephrine (1:100,000) was administered. After careful mucoperiosteal flap elevation, teeth were extracted to create defects. Mandibular third premolar (PM3) extractions facilitated flap mobilization to ensure tension-free wound closure, while extraction sockets corresponding to the mandibular fourth premolar (PM4) and the mesial root of the mandibular first molar (M1) were selected as experimental defect sites (Figure 1A).

Immediately following extraction, extraction sockets were then grafted with one of the three assigned experimental materials (HBC28, HBC37, HPC64). After graft material placement, sockets were covered with collagen membranes (OssGuide^®^, HYUNDAI BIOLAND, Republic of Korea), trimmed to half their original size, and closed with non-absorbable blue nylon sutures (AILEE Co., Busan, Republic of Korea).

Postoperative analgesia was provided using Meloxicam (Metacam, Boehringer Ingelheim, 0.1 mL/kg), while Cefovecin (Convenia, Zoetis Inc., Parsippany, NJ, USA, 8 mg/kg) was used for antibiotics.

### 2.5. Experimental Timeline and Evaluation Points

**Figure 2:**
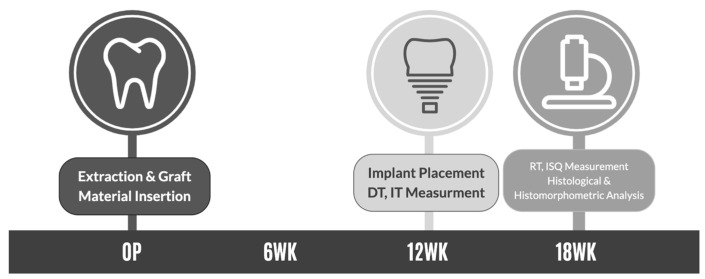
Experimental timeline outlining surgical interventions and evaluation points. At baseline (OP), teeth were extracted, and the defects were grafted with the assigned bone substitute materials. Implants (TSM3508S, Ø3.5 mm, length 8 mm, Osstem Implant Co., Ltd., Seoul, Repulic of Korea) were placed at 12 weeks post-extraction, with drilling torque (DT) and insertion torque (IT) measurements conducted at that time to assess primary stability. At 18 weeks post-extraction, removal torque (RT), implant stability quotient (ISQ), and histological and histomorphometric analyses were performed to evaluate secondary stability and the quality of bone regeneration.

The evaluation intervals and healing times were based on previously established histological and physiological timelines of bone remodeling in canine models. Considering the differences between human and canine bone remodeling rates, a healing period of 12 weeks (3 months) was established as appropriate for the evaluation of initial bone regeneration and stability. An additional 6-week period (18 weeks total) was chosen for the assessment of secondary stability and advanced remodeling (Figure 2).

Specifically, the experimental evaluation included the following:

- 12 weeks postoperation:
  ○ Implant placement (TSIII SA implants (Ø3.5 mm × 8.0 mm, OSSTEM IMPLANT, Seoul, Republic of Korea).
  ○ Measurement of drilling torque (DT) and insertion torque (IT) to assess primary stability and initial bone regeneration quality.
- 18 weeks post-operation:
  ○ Measurement of removal torque (RT) to evaluate the strength of bone-to-implant integration (secondary stability).
  ○ Implant stability quotient (ISQ) measurement using resonance frequency analysis (RFA) to objectively determine implant osseointegration and secondary stability.
  ○ Euthanasia and tissue harvesting for histological and histomorphometric analysis.

Implants were placed using an INTRAsurge 300 implant engine (KaVo, Biberach, Germany). IT and DT were measured with the implant engine, while RT measurements were performed using a digital torque driver (DTDK-N2EXL, KANON, Tokyo, Japan), and ISQ was assessed using an Osstell Beacon device (Osstell AB, Sweden).

### 2.6. Histological and Quantitative Analysis

After euthanasia at 18 weeks, mandibular block sections containing implants were carefully excised. Histological specimens were decalcified using 14% EDTA (pH 7.4) with daily replacement, embedded in Paraplast (Leica, Wetzlar, Germany), sectioned at 5 µm, and stained with hematoxylin–eosin.

**Figure 3:**
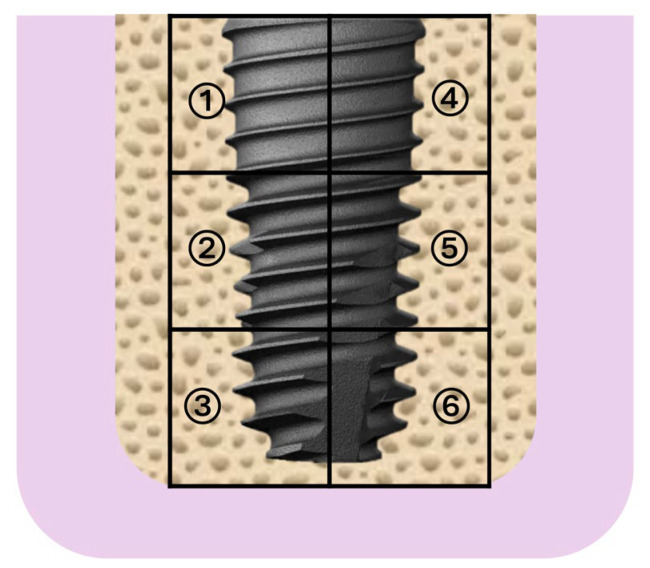
Illustration of the region of interest (ROI) for histological and histomorphometric analysis. The ROI was divided into six standardized areas around each implant for quantitative assessment of newly formed bone (BV/TV), residual graft material (RGV/TV), and soft tissue volume (ST/TV). Measurements from these regions ensured consistent and comparable histomorphometric analyses across different specimens and graft materials.

Bone regeneration outcomes among the three tested graft materials were assessed. Six standardized regions of interest (ROIs) surrounding each implant were precisely analyzed using image analysis software (ImageJ v1.53, NIH, Bethesda, MD, USA) to determine the following parameters (Figure 3):

New Bone Volume Fraction (BV/TV, %): Proportion of newly formed mineralized bone relative to total tissue volume, indicating bone regenerative capacity.

Residual Graft Volume Fraction (RGV/TV, %): Percentage of residual graft material remaining within the total tissue volume, reflecting the resorption rate and remodeling efficiency of graft materials.

Soft Tissue Fraction (ST/TV, %): Proportion of non-mineralized soft tissue within the ROI, indicative of fibrotic tissue infiltration and potential inflammation.

Bone Marrow Fraction (BM/TV, %): Quantitative measurement of the proportion of bone marrow spaces, representing regions of mature remodeling and marrow space formation following bone regeneration.

Six standardized ROIs around each implant were consistently selected, and data from these areas were averaged for statistical analysis.

### 2.7. Statistical Analysis

One-way analysis of variance (ANOVA) followed by Tukey’s post hoc test was applied for multiple group comparisons. All numerical data are expressed as mean ± standard deviation (SD). Statistical significance was set at *p* < 0.05.

## 3. Results

### 3.1. Surgical Outcome and Clinical Observations

All animals recovered uneventfully from surgery without significant complications. Wound closure was achieved without tension, and no dehiscence or graft exposure was observed during the healing period. The postoperative healing process was stable, and all implants were successfully placed after 12 weeks without intraoperative complications.

### 3.2. Mechanical Testing

**Figure 4:**
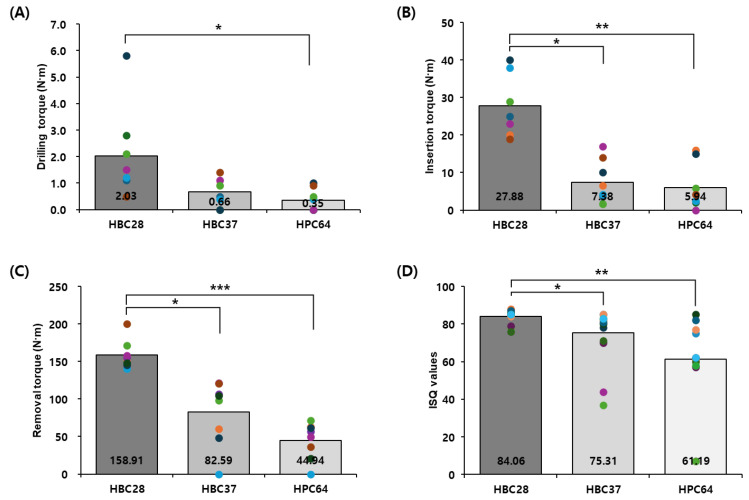
Mechanical performance of graft materials. (**A**) Drilling torque (DT) and (**B**) insertion torque (IT) at 12 weeks; (**C**) removal torque (RT) and (**D**) ISQ at 18 weeks. Bars represent means with individual data points; statistical significance: * *p* < 0.05, ** *p* < 0.01, *** *p* < 0.001.

Mechanical stability was assessed by measuring drilling torque (DT), insertion torque (IT), removal torque (RT), and implant stability quotient (ISQ). HBC28 exhibited higher mechanical values under the conditions tested across all timepoints and parameters (Table 2, Figure 4).

At 12 weeks, DT was significantly higher for HBC28 (2.03 ± 1.6 N·cm) compared to HBC37 (0.66 ± 0.5 N·cm, *p* = 0.1054) and HPC64 (0.35 ± 0.4 N·cm, *p* = 0.0181). Similarly, IT was markedly higher in HBC28 (27.88 ± 7.3 N·cm) than in HBC37 (7.38 ± 5.4 N·cm, *p* = 0.001) and HPC64 (5.94 ± 5.8 N·cm, *p* = 0.00027).

At 18 weeks, RT reached 158.91 ± 17.8 N·cm in the HBC28 group, significantly higher than HBC37 (82.59 ± 40.0 N·cm, *p* = 0.0004) and HPC64 (44.94 ± 22.8 N·cm, *p* < 0.00001). ISQ values further confirmed these trends, with HBC28 reaching 84.06 ± 3.3, followed by HBC37 (75.31 ± 13.9, *p* = 0.016) and HPC64 (61.19 ± 22.7, *p* = 0.0009).

### 3.3. Quantitative Histomorphometry

At 18 weeks post-grafting, HBC28 demonstrated the most favorable histomorphometric outcomes (Table 3). BV/TV was highest in HBC28 (51.23 ± 12.9%), followed by HBC37 (22.91 ± 9.2%) and HPC64 (12.47 ± 8.2%). RGV/TV values showed effective resorption in HBC28 (6.83 ± 6.3%), with higher residuals in HBC37 (21.95 ± 6.4%) and HPC64 (35.27 ± 8.3%).

ST/TV was lowest in HBC28 (4.08 ± 5.1%), while both HBC37 (47.55 ± 14.7%) and HPC64 (49.07 ± 10.4%) showed substantial fibrous infiltration. BM/TV values—indicative of advanced remodeling—were also most favorable in HBC28 (37.86 ± 11.6%) versus HBC37 (7.60 ± 8.6%) and HPC64 (3.19 ± 2.1%).

### 3.4. Histological Findings

**Figure 5:**
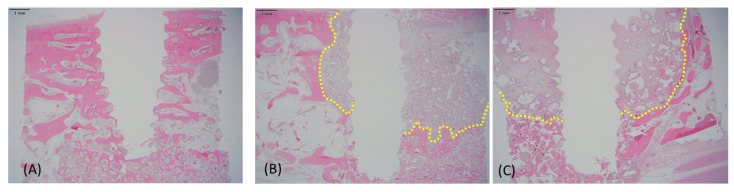
Representative histological images of graft-treated sites at 18 weeks post-grafting (hematoxylin and eosin staining; scale bar = 1 mm). (**A**) HBC28 showed robust formation of mature lamellar bone, minimal residual graft material, and limited fibrous tissue infiltration. (**B**) HBC37 exhibited moderate amounts of residual graft particles and delayed bone remodeling, with notable fibrous tissue presence. (**C**) HPC64 displayed limited new bone formation, extensive residual graft material, and substantial infiltration by fibrous tissue, indicative of slower remodeling and reduced regenerative efficiency. Residual graft materials are highlighted by yellow dashed outlines.

Histological evaluation at 18 weeks revealed prominent differences in bone remodeling among the three groups (Figure 5). HBC28-treated sites showed the extensive formation of mature lamellar bone, well-distributed marrow spaces, and minimal fibrous tissue infiltration. Residual graft particles in the HBC28 group were integrated into mature bone, with clear signs of remodeling.

Conversely, HBC37 and HPC64 showed delayed bone maturation and higher residual graft content. HPC64, in particular, exhibited the slowest remodeling, with large amounts of unresorbed graft particles and marked fibrous tissue infiltration. Although HBC37 performed moderately better than HPC64, both materials underperformed relative to HBC28.

These results support the conclusion that HBC28, with its optimized β-TCP-to-HA ratio and bovine collagen matrix, offers enhanced bone regeneration and remodeling characteristics.

## 4. Discussion

Clinically, immediate implant placement following tooth extraction has become common due to its efficiency and reduced treatment duration. However, immediate placement is not always feasible, particularly in cases involving significant infection, severe bone loss, or anatomical constraints. In these scenarios, socket preservation procedures are critical to maintaining alveolar ridge dimensions and preventing resorption that could compromise future implant placement.

Various graft materials have been employed clinically for socket preservation, including autogenous bone, xenografts, allografts, and synthetic grafts such as HA, β-TCP, and collagen composites [27,28]. Autogenous grafts, while effective, carry disadvantages of donor-site morbidity and limited availability [29]. Xenografts and allografts have inherent risks of disease transmission, immune reactions, and inconsistent resorption patterns [30]. Synthetic grafts like pure HA demonstrate very slow resorption, potentially hindering timely bone regeneration [31], while β-TCP alone might resorb too rapidly, risking inadequate volume maintenance [32]. Recent studies have focused on composite grafts combining these materials to improve outcomes [12,13,20]. However, few have directly compared clinically available β-TCP/HA/collagen composites with varied ratios and collagen origins under standardized in vivo conditions.

Among these, deproteinized bovine bone mineral (DBBM, e.g., Bio-Oss^®^) is one of the most widely used xenografts in clinical practice due to its excellent volume stability and long-term safety profile. However, DBBM is characterized by a very slow resorption rate, which may impede timely new bone replacement and compromise healing dynamics in cases requiring rapid remodeling [31,33]. In contrast, β-TCP-based synthetic materials offer faster resorption and remodeling, making them potentially more suitable for cases where early implant placement is desired. Therefore, this study focused on composite materials centered around β-TCP to investigate whether more favorable regenerative kinetics and mechanical integration could be achieved without sacrificing volumetric stability. In contrast to prior studies such as that of Maté-Sánchez de Val et al., which employed custom-fabricated HA/β-TCP/collagen scaffolds in rabbit calvarial critical-size defects, our study utilized commercially available materials in a canine mandibular extraction model—more closely replicating clinical conditions in human implantology [26]. Moreover, by incorporating both bovine and porcine collagen types and including clinically relevant metrics such as torque values and implant stability quotient (ISQ), this study offers new insight into how composite composition influences not only histological but also functional outcomes.

We examined the performance of three commercially available HA/β-TCP collagen composite grafts in a clinically relevant canine socket preservation model. Among the materials tested, HBC28 demonstrated greater new bone formation (BV/TV) and lower residual graft volume (RGV/TV) compared to HBC37 and HPC64. It also exhibited less fibrous tissue infiltration and fewer histological signs of inflammation, suggesting favorable integration and remodeling characteristics.

This study aimed to assess the comparative behavior of the bone grafts that clinicians may choose from in real-world practice. While all three materials showed clinically acceptable outcomes, HBC28 showed relatively more regenerative metrics under the conditions tested. Although HBC37 and HPC64 provide adequate initial structural support, their slower resorption and remodeling may delay healing and implant placement.

The performance difference between HBC28 and HBC37, despite similar compositional profiles, may be attributed to other contributing factors such as microstructural porosity and material-specific pH changes during dissolution—all of which can influence osteogenesis and cellular response [1,2]. A notable difference among the materials is the collagen content: HBC28 contains approximately 10% collagen, which is higher than that of HBC37 and HPC64 (approximately 4–6%). This difference in collagen concentration may influence biological response. Previous studies, such as that of Ferreira et al., have suggested that increased collagen content can enhance osteoconductivity, cellular attachment, or remodeling efficiency.

The composite nature of HBC28, with a balanced proportion of high-crystalline HA (20%) and higher β-TCP (80%) combined with bovine collagen, effectively addresses the drawbacks of the individual materials that make up the composite. The relatively lower HA content allows for controlled yet sufficient volume preservation during the early healing phases, while the higher proportion of β-TCP ensures faster remodeling conducive to timely implant placement. Collagen enhances graft stability, supports osteoconduction, and improves biological integration, further distinguishing HBC28 from other commercially available graft materials. Notably, HBC28 and HBC37 contain bovine collagen, whereas HPC64 incorporates porcine collagen. Differences in collagen source may affect bioresorption kinetics, cellular adhesion, and inflammatory response—potentially contributing to the differences observed in fibrous tissue infiltration among the groups. The apparent discrepancy between our findings and those of Maté-Sánchez de Val et al., where HA-rich scaffolds (60/20/20) showed superior outcomes, may be attributed to multiple variables. These include differences in collagen type and concentration, the animal model (rabbit calvaria vs. canine mandibular socket), mechanical loading conditions, and scaffold fabrication methods. Our use of commercially standardized materials in a functionally loaded mandibular site may have favored β-TCP-rich scaffolds (e.g., HBC28) due to their faster remodeling kinetics, whereas HA-dominant compositions may perform better in static, non-load-bearing environments such as the calvaria. These contextual factors should be considered when interpreting cross-study differences in bone regeneration outcomes. Further studies are warranted to isolate the effect of collagen origin on graft performance.

This study suggests that the optimization of the HA/β-TCP/collagen ratio may support consistent regeneration and remodeling in the context of socket preservation, ultimately improving outcomes in dental implantology. While bioactive materials such as BMP are frequently utilized, the development and adoption of cost-effective yet highly efficacious composite bone graft substitutes hold substantial clinical value.

Moreover, long-term clinical studies are necessary to assess its performance comprehensively across diverse patient populations and varying clinical conditions.

## 5. Conclusions

Within the limitations of this preclinical study, composite bone graft materials containing higher β-TCP content and moderate collagen proportions were associated with higher new bone formation and secondary implant stability compared to other grafts. Histomorphometric analysis confirmed increased bone marrow formation and reduced residual graft volume in the HBC28 group, supporting more efficient remodeling. These results support prior findings that β-TCP-rich materials enhance early remodeling, but also highlight the need to consider additional factors such as crystallinity, porosity, and collagen origin when designing graft composites. Optimizing the HA/β-TCP/collagen ratio is critical for enhancing graft performance in socket preservation procedures. While the current findings were obtained using a canine extraction socket model, future studies—particularly randomized clinical trials in humans—are necessary to validate the clinical applicability and long-term outcomes of these materials.

## Figures and Tables

**Table 1:** Composition and characteristics of bone graft materials used in this study.

Material	HBC28	HBC37	HPC64
HA ^1^ (%)	20	30	60
β-TCP ^1^ (%)	80	70	40
Collagen Source ^1^ (%)	Bovine type 1 (10%)	Bovine type 1 (4–6%)	Porcine type 1 (6%)
Additional Features	Micron-sized crystal	Porosity 70%	Porosity 80%
Microcrystal Structure ^2^	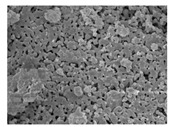	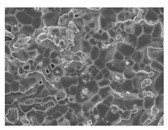	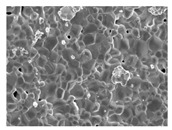
Collagen Attachment to Graft Material ^2^	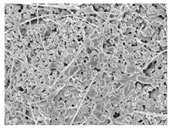	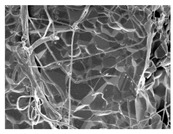	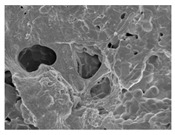

^1^ HA and β-TCP percentages represent the ceramic phase composition. Collagen content is added separately by weight. ^2^ Scanning electronic electroscope (SEM) image at 5 k resolution.

**Table 2:** Mechanical stability measurements for each graft material.

Material	DT (N) ^1^	IT (N) ^1^	RT (N) ^2^	ISQ ^2^	*p*-Value vs. HBC28 (DT)	*p*-Value vs. HBC28 (IT)	*p*-Value vs. HBC28 (RT)	*p*-Value vs. HBC28 (ISQ)
HBC28	2.03 ± 1.6	27.88 ± 7.3	158.91 ± 17.8	84.06 ± 3.3	—	—	—	—
HBC37	0.66 ± 0.5	7.38 ± 5.4	82.59 ± 40.0	75.31 ± 13.9	0.1054	0.001	0.0004	0.016
HPC64	0.35 ± 0.4	5.94 ± 5.8	44.94 ± 22.8	61.19 ± 22.7	0.0181	0.00027	<0.00001	0.0009

DT: mean drilling torque, IT: mean insertion torque, RT: mean removal torque, ISQ: implant stability quotient. ^1^ Measurements conducted at the time of implant placement (12 weeks post-grafting). ^2^ Measurements performed at 18 weeks post-grafting.

**Table 3:** Quantitative analysis results at 18 weeks post-grafting (mean ± SD).

Material	BV/TV (%)	RGV/TV (%)	ST/TV (%)	BM/TV (%)
HBC28	51.23 ± 12.9	6.83 ± 6.3	4.08 ± 5.1	37.86 ± 11.6
HBC37	22.91 ± 9.2	21.95 ± 6.4	47.55 ± 14.7	7.60 ± 8.6
HPC64	12.47 ± 8.2	35.27 ± 8.3	49.07 ± 10.4	3.19 ± 2.1

BV/TV: mean bone volume fraction, RGV/TV: Residual Graft Volume Fraction, ST/TV: Soft Tissue Fraction, BM/TV: Bone Marrow Fraction.

## Data Availability

The data presented in this study are available on request from the corresponding author. The data are not publicly available due to the institutional policy.

## Abbreviations

The following abbreviations are used in this manuscript:

HA	Hydroxyapatite
β-TCP	Beta-Tricalcium Phosphate
BV/TV	Bone Volume to Total Volume
RGV/TV	Residual Graft Volume to Total Volume
ST/TV	Soft Tissue to Total Volume
BM/TV	Bone Marrow to Total Volume
ISQ	Implant Stability Quotient
DT	Drilling Torque
IT	Insertion Torque
RT	Removal Torque
ROI	Region of Interest
